# Advances in molecular imaging: diagnostic, technical, and therapeutic considerations—introductory Editorial

**DOI:** 10.1093/bjr/tqag123

**Published:** 2026-05-28

**Authors:** Patrick Veit-Haibach, Cristina Nanni

**Affiliations:** Toronto Joint Department Medical Imaging, University Medical Imaging Toronto, Toronto, ON, M5G 2N2, Canada; University Health Network, Sinai Health System, Women’s College Hospital, Toronto, ON, M5G 2N2, Canada; Department of Medical and Surgical Sciences, University of Bologna, Via Zamboni, 33, 40126 Bologna BO, Italy

This *BJR* special feature captures a potential inflection point in nuclear medicine, where technological innovation, radiopharmaceutical development, and computational advances are converging and are redefining precision imaging and theranostics. Multiple topics, ranging from new technologies, the newest and most promising tracers, as well as other non-medical but equally important issues like workforce sustainability are discussed and put in perspective of the current literature and molecular imaging landscape.

In the first contribution, Karakatsanis et al. explain the fundamental transformation that arrived with long axial field-of-view (LAFOV) PET, a platform that significantly alters how we acquire, quantify, and interpret molecular data across the entire human body.^1^ LAFOV PET systems, with axial coverage extending up to 200 cm, deliver sensitivity not seen before in clinical imaging and enable simultaneous whole-body dynamic imaging. This capability is not merely incremental but enables true multi-parametric imaging at scale, where tracer kinetics, organ interplay, and systemic disease biology can be evaluated in a single acquisition. Such advances position LAFOV PET as a cornerstone for next-generation applications, including quantitative response assessment, ultra-low-dose imaging paradigms, and the emerging concept of theranostic “digital twins,” where individualized dosimetry and treatment simulation become clinically actionable.

Complementing these hardware advances is a rapidly expanding portfolio of radiopharmaceuticals. While fibroblast activation protein (FAP)-targeted imaging has emerged as a dominant paradigm—demonstrating superior lesion detectability and growing theranostic applicability—the field is broadening significantly. Novel agents targeting estrogen receptors, CXCR4, carbonic anhydrase IX, and somatostatin receptor antagonists illustrate the increasing biological diversity of actionable targets. This diversification reflects a shift from single-pathway imaging toward a more nuanced interrogation of tumor biology and microenvironmental complexity.^2^^,^^3^

Parallel developments in SPECT/CT and PET/MR further reinforce the multi-parametric imaging landscape. Modern SPECT/CT, leveraging digital detectors and quantitative reconstruction, is re-establishing itself as a critical modality for dosimetry and radionuclide therapy assessment, particularly given its compatibility with long-lived isotopes and multi-tracer studies.^4^ PET/MR, in turn, offers unparalleled soft-tissue characterization and integrated functional imaging, with advances in motion correction, attenuation modeling, and dynamic quantification enhancing its role in both research and select clinical applications.^5^

Importantly, these imaging innovations are tightly coupled and needed for a rapidly evolving global theranostic pipeline. Clinical trials targeting PSMA, GRPR, and FAP are advancing through late-phase development, with several poised to influence clinical practice within the next 5 years. The development toward multi-targeted and combination theranostic strategies reflects an acknowledgment of tumor heterogeneity and resistance mechanisms, underscoring the need for integrative imaging biomarkers.^6^

No current state-of-the-art publication is currently thinkable with discussing artificial intelligence (AI). AI emerges as a critical enabler across molecular imaging and theranostic ecosystem. From accelerating image acquisition and improving reconstruction to enabling automated interpretation and predictive modeling, AI has the potential to bridge the gap between technological capability and clinical scalability. It might also help to address some of the workforce, workflow, and workload issues that seem to arise globally in healthcare. However, despite substantial promise, clinical translation remains limited, highlighting the need for robust validation and implementation frameworks.^7^

Finally, the rapid expansion of theranostics brings into focus a pressing workforce challenge. The global shortage of trained specialists necessitates coordinated efforts in education and training to ensure equitable access to these advanced therapies.^8^

Together, the contributions in this *BJR* special feature underscore a central thesis: the future of nuclear medicine lies in integrated, quantitative, personalized, and biologically driven imaging ecosystems. Implementation of this vision on a global scale will require not only continued innovation but also strategic efforts to ensure accessibility, scalability, and clinical adoption worldwide.
